# Effect of disappearing liver metastases during pre-ablation chemotherapy on the prognosis of percutaneous microwave ablation in synchronous colorectal liver metastases patients

**DOI:** 10.1186/s40644-025-00943-4

**Published:** 2025-10-28

**Authors:** Limei Chen, Jingwen Zhou, Rui Cui, Si Qin, Yao Chen, Yimin Wang, Guangjian Liu

**Affiliations:** 1 Department of Ultrasonography, The Sixth Affiliated Hospital, Sun Yat-sen University, Guangzhou, Guangdong China; 2 Biomedical Innovation Center, The Sixth Affiliated Hospital, Sun Yat-sen University, Guangzhou, Guangdong China

**Keywords:** Colorectal liver metastases, Chemotherapy, Microwave ablation, Ultrasound, Survival

## Abstract

**Introduction:**

Disappearing colorectal liver metastases (DLM) frequently occur during chemotherapy. However, DLM is not equivalent to pathologically complete response. This study aimed to investigate the effect of radiographic DLM on microwave ablation (MWA) in patients with synchronous colorectal liver metastases (CRLM).

**Methods:**

A retrospective review was performed for patients who accepted MWA following pre-ablation chemotherapy from January 2014 to December 2021. DLM was defined as undetectable tumors on pre-ablation contrast-enhanced imagings compared to the initial ones. Overall survival (OS) and intrahepatic progression-free survival (ihPFS) were analyzed and compared between patients with and without DLM. Univariate and multivariate cox regression were used to identify risk factors for OS and ihPFS. A propensity score matching (PSM) analysis was used to balance the patient demographics.

**Results:**

Sixty-eight patients with DLM and 97 without DLM were included. The 1-year, 3-year, and 5-year ihPFS rates were significantly lower for patients with DLM compared to those without DLM before and after PSM (55.7%, 36.8%, and 30.6% vs. 70.8%, 59.3%, and 52.0% before PSM, respectively, *p* = 0.012; 44.9%, 31.8%, and 21.2% vs. 72.3%, 58.8%, and 47.5% after PSM, respectively, *p* = 0.039). Twenty-three (33.8%) patients with DLM had DLM-site recurrences during follow-up. The OS was not statistically different between the two groups both before and after PSM (p-value = 0.11 and 0.49). Multivariable cox regression revealed DLM (HR = 2.2; 95% CI = 1.1–4.1; p-value = 0.009) was a risk factor for poor ihPFS.

**Conclusion:**

Patients with DLM presented worse ihPFS, suggesting that to eradicate visible tumors before disappearance may be advantageous when synchronous CRLM is ablatable.

## Introduction

Synchronous colorectal liver metastases (sCRLM) are identified concurrently with the diagnosis of colorectal cancer, occurring in approximately 20% of patients with this malignancy [1]. The presence of sCRLM is associated with a more aggressive cancer biology and is generally linked to poorer survival outcomes compared to metachronous metastases [2, 3].

Currently, systemic chemotherapy is extensively administered to patients with sCRLM. For resectable sCRLM, up-front surgery remains the mainstream treatment, but chemotherapy before surgery has markedly extended disease survival in these patients [4, 5], especially for those with high tumor burden and risk of poor prognosis. For unresectable sCRLM, preoperative chemotherapy is offered with the goal of downsizing tumors to allow local treatment [6–8]. However, with the advance of chemotherapy, the phenomenon of disappearing colorectal liver metastases (DLM) is becoming increasingly common. Radiographic DLM is reported in 7%−48% of patients receiving chemotherapy [9], yet it does not necessarily equal to a pathologically complete remission. Studies indicated that approximately 15% to 61% of the radiographic DLM showed pathologically viable cancer cells or recurred during the follow-up [9–12].

Imaging-guided thermal ablation is widely used to treat patients with liver malignant tumors for over 20 years, with the advantage of minimal invasiveness and repeatable nature. The combined use of chemotherapy and local treatment significantly improve recurrence-free survival as well as overall survival (OS) compared to those who received chemotherapy alone [13, 14]. However, the optimal timing for neoadjuvant chemotherapy and local treatment remains an undetermined problem. Had radiographic DLM occurs during chemotherapy, percutaneous thermal ablation is limited by the resolution of imaging modalities in eliminating the micro metastases at DLM sites. Therefore, once DLM occurs during chemotherapy and is left untreated due to percutaneous approach, these patients may lose the opportunity of achieving no evidence of disease (NED).

The impact of DLM on the outcomes of MWA for patients with sCRLM remains inadequately understood. We hypothesized that DLM could negatively impact the prognosis of MWA, leading to different survival outcomes for patients with DLM compared to those without. The aim of this study was to determine the effect of DLM on MWA by comparing survival outcome of sCRLM patients with or without DLM.

## Methods and materials

### Study design

This retrospective study was endorsed by the Institution Ethics Committee (approved number: 2024ZSLYEC-171) and adhered to the Declaration of Helsinki and the International Conference on Harmonization-Good Clinical Practice. This retrospective study examined consecutive patients treated for synchronous CRLM at a high-volume colorectal cancer center from January 2014 to December 2021. Inclusion criteria included: 1. Diagnosis of sCRLM confirmed by at least two contrast imaging methods or liver biopsy; 2.Pathologically confirmed colorectal cancer in which the primary tumors were radically resected; 3. Patients who received pre-ablative chemotherapy without disease progression; 4. Patients who had fewer than five tumors and a maximum tumor size of 3 cm who received technically successful ultrasound-guided MWA following pre-ablative chemotherapy; 5. Availability of contrast imaging data both prior and following pre-ablative chemotherapy; 6. Regular follow-up with contrast imaging for a minimum of six months. Exclusion criteria were: (1) Presence of other primary malignant tumors; (2) Concurrent hepatectomy or transarterial chemoembolization with MWA; (3) Occurrence of new CRLM during pre-ablative chemotherapy.

According to the CRLM visualization on pre- and post- chemotherapy, patients were classified into those with DLM and without DLM. DLM was defined as the absence of tumors on pre-MWA contrast-enhanced imaging (CECT *n* = 125, CEMRI *n* = 53) compared to the initial ones. The survival outcomes and recurrence patterns were compared between patients with DLM and those without. Recurrences were categorized as either in situ or de novo. In situ recurrence included local tumor progression (LTP) and recurrence at the DLM site. LTP was identified by the presence of a hyper-enhanced nodule with washout at the edge of the ablated zone after complete ablation, as seen in contrast-enhanced imaging studies. DLM site recurrence was characterized by the presence of metastases visible on CECT or CEMRI prior to chemotherapy that subsequently disappeared following chemotherapy. De novo recurrence referred to new tumors appearing at locations distant from the initial site of metastases, identified on pre-chemotherapy imaging scans. Intrahepatic progression-free survival (ihPFS) was defined as the duration from the date of MWA to intrahepatic tumor recurrence or the last follow-up. Overall survival (OS) was defined as the duration from the date of MWA to the date of death or the last follow-up.

### Treatment strategy

All patients underwent chemotherapy prior to percutaneous US-guided MWA, with the chemotherapy regimens and cycles determined by the oncologists at our hospital. After chemotherapy, a multidisciplinary team—including an experienced medical oncologist, radiation oncologist, gastrointestinal surgeon, hepatobiliary surgeon, radiologist, interventionist, and pathologist—evaluated the feasibility of ablating sCRLM. Patients with a maximum of five tumors and tumor size no larger than 3 cm, were eligible for percutaneous US-guided MWA.

Before performing MWA, ultrasound examination with convex probe was performed to detect CRLM initially. We read the pre- and post-chemotherapy CECT or CEMRI for reference. Once lesions on pre-chemotherapy could not be detected by ultrasound scanning with convex probe, high-frequency ultrasound and contrast enhanced ultrasound (CEUS) using linear probe were combined with conventional convex probe US and CEUS to maximize the detection of tumors after chemotherapy [15]. All identified lesions were treated with MWA, while DLMs were unablated and no anatomical orientated approach was applied. The MWA procedures utilized a 2450-MHz microwave ablation system (KY2000; Nanjing Kangyou Biological Energy Co. Ltd, Nanjing, China), as previously detailed by our center [16, 17]. In brief, a 15 G dual-channel antenna was positioned 5 mm beyond the deep margin of the lesion under ultrasound guidance (LOGIQ E9; GE Healthcare, Milwaukee, WI, USA). The ablation strategies depended on the size of the lesions: a single antenna was used for lesions ≤ 2.0 cm, while 1–2 antennas were applied for lesions measuring 2.0–3.0 cm, with energy output adjusted between 45 and 60 W for 5–15 min. Throughout the procedure, the temperature of each antenna was kept below 40 °C by circulating cold saline. The needle track was also ablated to reduce the risk of bleeding and tumor seeding. Each procedure was conducted by the same interventionist, who has over 20 years of experience in liver cancer ablation.

Since all MWA procedures in the recruited patients were performed via a percutaneous approach, simultaneous open or laparoscopic MWA during colorectal cancer resection was not conducted. In this study, three timing strategies for MWA and colorectal resection in sCRLM patients were defined. The concurrent strategy was characterized by an interval of ≤ 14 days between MWA treatment of sCRLM and resection of the primary tumor. The bowel-first approach involved initial resection of the primary lesion followed by liver ablation after an interval of > 14 days. Conversely, the liver-first approach entailed MWA of liver metastases followed by resection of the primary tumor after an interval exceeding 14 days.

### Follow-up

CEUS was performed 30 min and 24 h after MWA, followed by CEUS and CECT/MRI one-month post-MWA. Recurrence and survival data were then monitored using contrast-enhanced imaging techniques every three months for the first six months and every six months thereafter for follow-up according to the standard care protocol for CRLM patients of our center.

### Statistical analysis

Categorical variables were presented as counts (percentages), while continuous variables were summarized as median (1st quartile, 3rd quartile) or mean ± standard deviation. The chi-squared test was employed to compare categorical data, and the T-test was utilized for the comparison of continuous data. Survival outcomes, including ihPFS and OS, were analyzed using Kaplan–Meier methods and compared with the log-rank test. Univariate and multivariate Cox regression analyses were conducted to identify significant factors associated with ihPFS and OS. Variables with a p-value less than 0.2 in univariate analysis were considered eligible for inclusion in the multivariable Cox regression. Multivariable Cox proportional hazards regression was then performed to obtain adjusted hazard ratios (HRs) and 95% confidence intervals (CIs) for ihPFS and OS. To reduce the impact of baseline differences in demographic, the 1:1 propensity score matching (PSM) method was used to match the patients with and without DLM. The PSM method confirms background differences by standardized differences. A p-value of less than 0.05 was deemed statistically significant. Statistical analyses were conducted using IBM SPSS Statistics version 25.0 (International Business Machines Corporation, New York) and R Statistical Software version 4.3 (Foundation for Statistical Computing, Vienna, Austria).

## Results

### Patient demographics

A total of 165 patients with sCRLM who met the inclusion criteria were included in this study. After pre-ablative chemotherapy, 68 patients had at least one DLM and 97 showed no DLM. Of 165 patients, 32 (19.4%) underwent a liver-first approach, 69 (41.8%) a concurrent approach and 64 (38.8%) a bowel-first approach. The patient demographics of patients with and without DLM were summarized in Table 1. The median follow-up period was 48.2 months (95% CI: 41.9–54.5 months). The median age in patients with and without DLM were 60 (53, 65) and 58 (52, 64), respectively. Patients with more than two sCRLM at the time of diagnosis and more than two sCRLM at the time of MWA were found more prevalent in patients with DLM than those without DLM (*p* < 0.001 and *p* = 0.010, respectively). No significant differences were observed in other factors between the two groups (all *p* > 0.050).

**Table 1:** Demographics of patients with and without DLM before propensity score matching

Characters	Patients without DLM (*n* = 97)	Patients with DLM (*n* = 68)	*p*-value
**Age**,** median(quartile)**	58 (52,64)	60(53,65)	0.180
**Gender (%)**			1.000
Female	26.8% (26/97)	27.9% (19/68)	
Male	73.2% (71)	72.1% (49/68)	
**Plasma CEA (%)**			0.282
≤10 µg/L	77.3% (75/97)	85.3% (58/68)	
>10 µg/L	22.7% (22/97)	14.7% (10/68)	
**Primary cancer**			
**Location (%)**			1.000
Left	88.7% (86/97)	88.2% (60/68)	
Right	11.3% (11/97)	11.8% (8/68)	
**Pathologic tumor category (%)**			1.000
ypT0 – ypT3	82.5% (80/97)	82.4% (56/68)	
ypT4	17.5% (17/97)	17.7% (12/68)	
**Pathologic lymphatic spread (%)**			0.317
N0	30.9% (30/97)	39.7% (27/68)	
N1 or N2	69.1% (67/97)	60.3% (41/68)	
**Extrahepatic metastasis (%)**			0.522
No	76.3% (74/97)	70.6% (48/68)	
Yes	23.7% (23/97)	29.4% (20/68)	
**Initial CRLM**			
**Number (%)**			**< 0.001**
≤ 2	77.3% (75/97)	16.2% (11/68)	
> 2	22.7% (22/97)	83.8% (57/68)	
**Maximum diameter (%)**			0.885
≤ 3 cm	74.2% (72/97)	76.5% (52/68)	
> 3 cm	25.8% (25/97)	23.5% (16/68)	
**Ablated CRLM**			
**Number (%)**			**0.010**
≤ 2	77.3% (75/97)	57.4% (39/68)	
3–5	22.7% (22/97)	42.6% (29/68)	
**Maximum diameter (%)**			0.428
≤ 16 mm	64.9% (63/97)	72.1% (49/68)	
> 16 mm, ≤ 30 mm	35.1% (34/97)	27.9% (19/68)	
**Distribution (%)**			0.716
unilobar	71.1% (69/97)	66.2% (45/68)	
bilobar	28.9% (28/97)	33.8% (23/68)	
**Timing of MWA for sCRLM**			0.354
Bowel-first	39.2% (38/97)	38.2% (26/68)	
Liver-first	22.7% (22/97)	14.7% (10/68)	
Concurrent	38.1% (37/97)	47.1% (32/68)	

*DLM* Disappearing colorectal liver metastases, *CRLM* Colorectal liver metastases, *CEA* Carcinoembryonic antigen, *MWA* Microwave ablation

The characteristics of pre-ablative chemotherapy were listed in Table 2. Patients with DLM underwent more cycles of pre-ablative chemotherapy than those without DLM (median cycles: 6 (4, 8) vs. 4 (3, 6), *p* < 0.001). There were no significant differences regarding the pre-ablative chemotherapy regimens used.

**Table 2:** Characteristics of pre-ablative chemotherapy

Characteristics	Patients without DLM	Patients with DLM	*P*-value
Number of chemotherapy cycles Median (quartile)	4(3,6)	6(4,8)	< 0.001
Chemotherapy regimens used			0.957
Oxaliplatin-based	64	43	
Oxaliplatin &Irinotecan	31	23	
Other	3	2	

*DLM* Disappearing colorectal liver metastases

The 1:1PSM analysis was also performed between the groups of patients to reduce the impact of bias. Ultimately, 66 patients were assessed and each subgroup included 33patients. The *P*-values for all covariates were greater than 0.05, indicating that propensity scores for the two groups significantly overlapped (Table 3).

**Table 3:** Demographics of patients with and without DLM after propensity score matching

Characters	Patients without DLM (*n* = 33)	Patients with DLM (*n* = 33)	*p*-value
**Age**			0.806
**≤60**	48.5% (16/33)	51.5% (17/33)	
**>60**	51.5% (17/33)	48.5% (16/68)	
**Gender (%)**			0.100
Female	36.4% (12/33)	18.2% (6/33)	
Male	63.6% (21/33)	81.8% (27/33)	
**Plasma CEA (%)**			1.000
≤10 µg/L	81.8% (27/33)	81.8% (27/33)	
>10 µg/L	18.2% (6/33)	18.2% (6/33)	
**Primary cancer**			
**Location (%)**			0.672
Left	87.9% (29/33)	93.9% (31/33)	
Right	12.1% (4/33)	6.1% (2/33)	
**Pathologic tumor category (%)**			1.000
ypT0 – ypT3	84.8% (28/33)	84.8% (28/33)	
ypT4	15.2% (5/33)	15.2% (5/33)	
**Pathologic lymphatic spread (%)**			0.605
N0	30.3% (10/33)	39.4% (13/33)	
N1 or N2	69.7% (23/33)	60.6% (20/33)	
**Extrahepatic metastasis (%)**			0.605
No	69.7% (23/33)	60.6% (20/33)	
Yes	30.3% (10/33)	39.4% (13/33)	
**Initial CRLM**			
**Number (%)**			1.000
≤ 2	33.3% (11/33)	33.3% (11/33)	
> 2	66.7% (22/33)	66.7% (22/33)	
**Maximum diameter (%)**			0.789
≤ 3 cm	66.7% (22/33)	72.7% (24/33)	
> 3 cm	33.3% (11/33)	27.3% (9/33)	
**Ablated CRLM**			
**Number (%)**			1.000
≤ 2	66.7% (22/33)	66.7% (22/33)	
3–5	33.3% (11/33)	33.3% (11/33)	
**Maximum diameter (%)**			1.000
≤ 16 mm	30.3% (10/33)	30.3% (10/33)	
> 16 mm, ≤ 30 mm	66.7% (23/33)	66.7% (23/33)	
**Distribution (%)**			0.622
unilobar	48.5% (16/33)	57.6% (19/33)	
bilobar	51.5% (17/33)	42.4% (14/33)	

*DLM* Disappearing colorectal liver metastases. *CRLM* Colorectal liver metastases. *CEA* Carcinoembryonic antigen

### Intrahepatic recurrence free survival for patients with and without DLM

During follow-up, intrahepatic disease progression occurred in 79.4% (54/68) and 45.4% (44/97) of patients with and without DLM, respectively (*p* < 0.001). The recurrence patterns for patients with and without DLM were shown in Table 4. For patients with DLM, in situ recurrence occurred in 31 patients (including 8 LTP, 23 DLM site recurrences). For patients without DLM, in situ recurrence occurred in 13 patients who developed LTP. The in situ recurrence rate was significantly higher for patients with DLM than those without DLM (45.6% (31/68) versus 13.4% (13/97), *p* < 0.001). There was no statistical difference of de novo recurrence between two groups (33.8% (23/68) versus 31.9% (31/97), *p* = 0.802).

**Table 4:** Intrahepatic recurrence patterns of patients with or without DLM

Recurrence patterns	Patients without DLM	Patients with DLM	*P*-value
Recurrence insitu (%)	13.4% (13/97)	45.6% (31/68)	< 0.001
LTP (n)	13	8	
DLM site recurrence (n)	NA	23	
Recurrence de novo (%)	31.9% (31/97)	33.8% (23/68)	0.802

*DLM* Disappearing colorectal liver metastases, *LTP* Local tumor progression

**Fig. 1:**
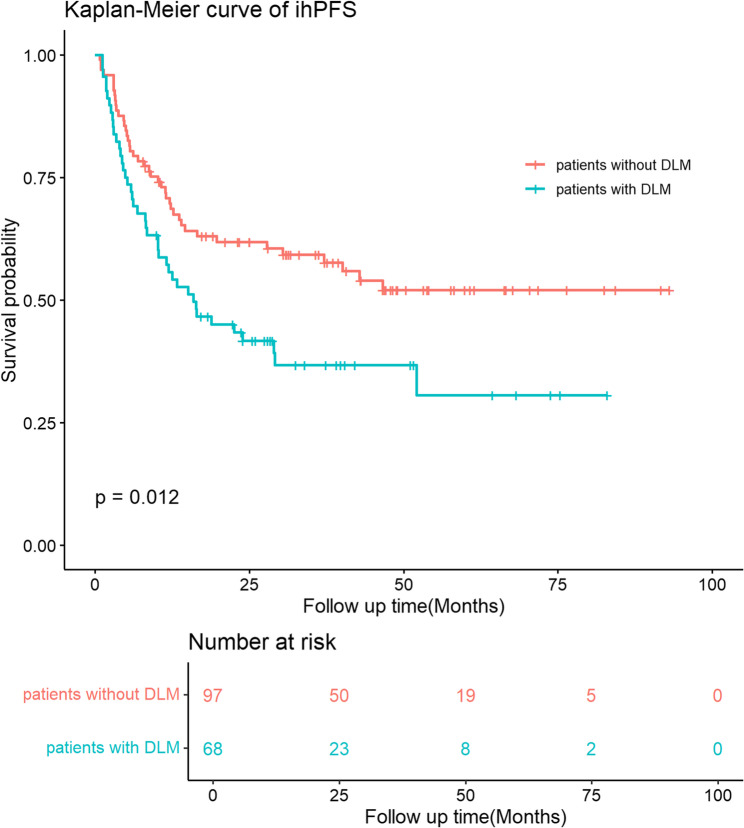
ihPFS of patients with and without DLM before propensity score matching

**Fig. 2:**
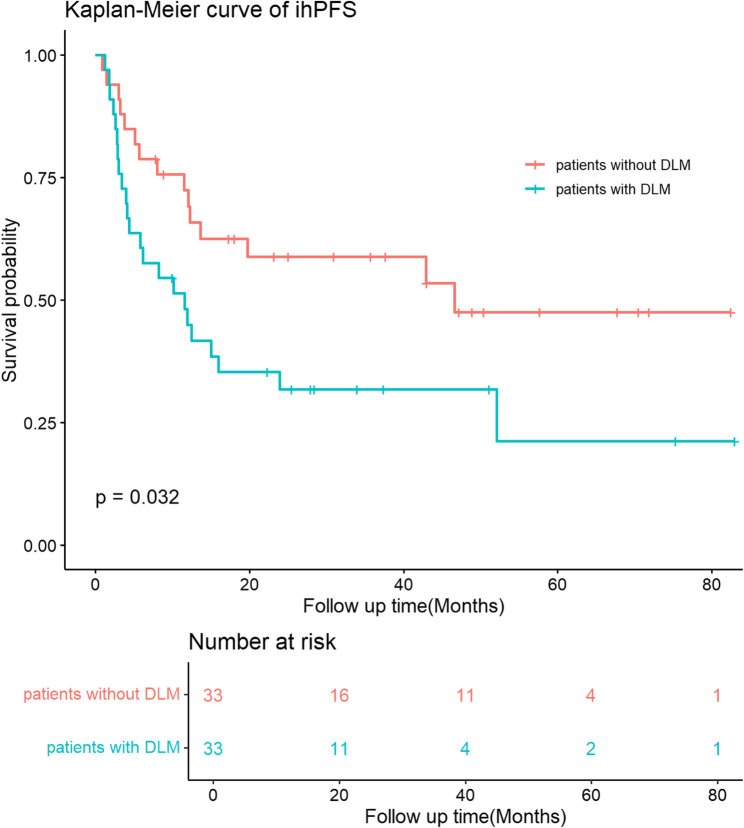
ihPFS of patients with and without DLM after propensity score matching

Before PSM, the 1-year, 3-year, and 5-year ihPFS rates for patients with DLM were 55.7%, 36.8%, and 30.6%, respectively, which is significantly lower than patients without DLM whose 1-year, 3-year, and 5-year ihPFS were 70.8%, 59.3%, and 52.0%, respectively. (p-value = 0.012, Fig. 1). After PSM, the ihPFS rates for patients with DLM were 44.9%, 31.8%, and 21.2% at 1-year, 3-year, and 5-year, respectively, which is significantly lower than patients without DLM whose 1-year, 3-year, and 5-year ihPFS were 72.3%, 58.8%, and 47.5%, respectively. (p-value = 0.039, Fig. 2).

On univariable cox regression, extrahepatic metastasis (HR: 1.7; 95% CI: 1.1–2.6; *p* = 0.029), ablated tumor number (HR: 1.6; 95% CI: 1.0–2.5.0.5; *p* = 0.036), and DLM (HR: 1.7; 95% CI: 1.1–2.7; *p* = 0.013) were identified as three independent factors associated with poor ihPFS. On multivariable analyses, extrahepatic metastasis (HR: 1.8; 95% CI: 1.1–2.8; *p* = 0.018), T4 stage primary cancer (HR: 1.8; 95% CI: 1.0–3.0; *p* = 0.043), tumor size > 16 mm (HR: 1.9; 95% CI: 1.2–3.0.2.0; *p* = 0.007), and DLM (HR: 2.2; 95% CI: 1.1–4.1; *p* = 0.009) were identified as risk factors associated with ihPFS (Table 5).

**Table 5:** Univariate and multivariate Cox regression for IhPFS in sCRLM patients received pre-ablative chemotherapy

Characters	Category	*N*	Univariate analysis		Multivariate analysis	
			HR (95%CI)	*p*-value	HR (95%CI)	*p*-value
**Age**	≤ 60	92	Reference			
	> 60	73	1.1 (0.7–1.7)	0.606		
**Gende**r	Female	45	Reference			
	Male	120	1.1 (0.7–1.8)	0.636		
**Plasma CEA**	≤ 10 µg/L	133	Reference			
	> 10 µg/L	32	0.7 (0.4–1.3)	0.289		
**Primary cancer**						
**Location**	Left	146	Reference			
	Right	19	0.9 (0.5–1.9)	0.905		
**Pathologic tumor category**	ypT0 – ypT3	136	Reference			
	ypT4	29	1.5 (0.9–2.6)	0.139	1.8 (1.0–3.0)	**0.043**
**Pathologic lymphatic spread**	Negative	57	Reference			
	Positive	108	1.2 (0.7–1.8)	0.522		
**Extrahepatic metastasis**	No	122	Reference			
	Yes	43	1.7 (1.1–2.6)	**0.029**	1.8 (1.1–2.8)	**0.018**
**Initial CRLM**						
Number	≤ 2	86	Reference			
	> 2	79	1.4 (0.9–2.2)	0.095	0.6 (0.3–1.3)	0.190
Maximum diameter	≤ 3 cm	124	Reference			
	> 3 cm	41	1.2 (0.7–1.9)	0.493		
**Ablated CRLM**						
Number	≤ 2	114	Reference			
	> 2	51	1.6 (1.0–2.5.0.5)	**0.036**	1.8 (0.9–3.5)	0.074
Maximum diameter	≤ 16 mm	112	Reference			
	> 16 mm, ≤ 30 mm	53	1.6 (0.9–2.4)	0.055	1.9 (1.2–3.0.2.0)	**0.007**
Distribution	unilobar	113	Reference			
	bilobar	52	1.1(0.7–1.7)	0.768		
**DLM**	No	97	Reference			
	Yes	68	1.7 (1.1–2.7)	**0.013**	2.2 (1.1–4.1)	**0.009**
**Timing of MWA for sCRLM**	Bowel-first	64	Reference			
	Liver-first	32	0.8 (0.5–1.6)	0.602		
	Concurrent	69	1.2 (0.7–1.9)	0.530		

*DLM* disappearing colorectal liver metastases, *CRLM* colorectal liver metastases, *CEA* carcinoembryonic antigen

### Overall survival for patients with and without DLM

**Fig. 3:**
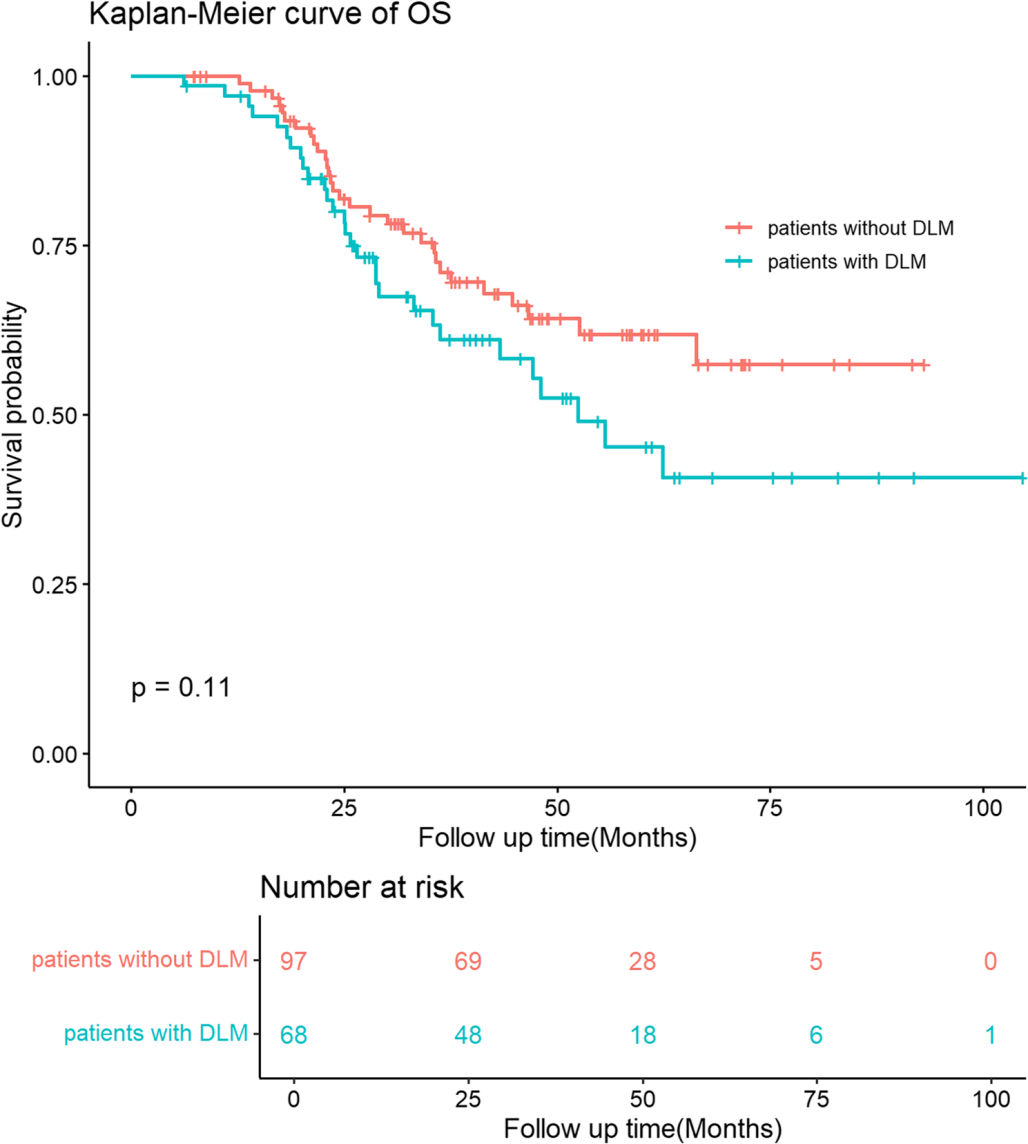
OS of patients with and without DLM before propensity score matching

**Fig. 4:**
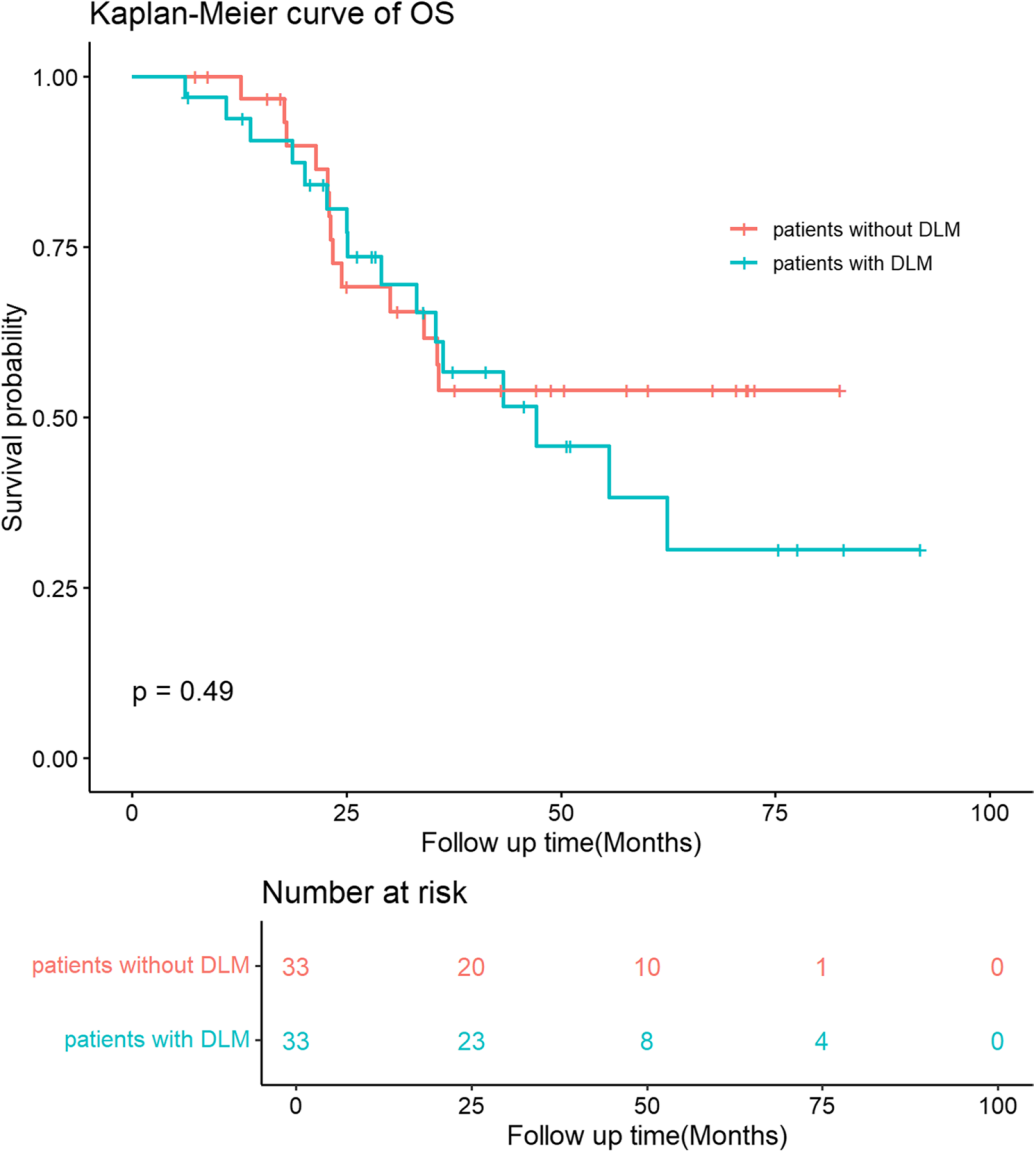
OS of patients with and without DLM after propensity score matching

Before PSM, the difference of OS was not statistically significant between patients without and with DLM (100% vs. 97.0%, 72.5% vs. 63.3%, and 61.8% vs. 45.2% for 1-year, 3-year and 5-year OS, respectively, p-value = 0.11, Fig. 3). After PSM, the difference of OS was still not statistically significant between patients without and with DLM (100% vs. 93.8%, 53.9% vs. 61.1%, and 53.9% vs. 38.2% for 1-year, 3-year and 5-year OS, respectively, p-value = 0.49, Fig. 4).

On univariable cox regression, age over 60 (HR: 2.0; 95% CI: 1.2–3.3; *p* = 0.009) and right colon cancer (HR: 2.5; 95% CI: 1.3–5.0.3.0; *p* = 0.008) were identified as independent factors associated with poor OS. On multivariable analyses, age over 60 (HR: 2.3; 95% CI: 1.4–4.0.4.0; *p* = 0.002), right colon cancer (HR: 2.5; 95% CI: 1.2–5.2; *p* = 0.011) were identified as risk factors associated with OS (Table 6).

**Table 6:** Univariate and multivariate Cox regression for OS in sCRLM patients received pre-ablative chemotherapy

Characters	Category	*N*	Univariate analysis		Multivariate analysis	
			HR (95%CI)	*p*-value	HR (95%CI)	*p*-value
**Age**	≤ 60	92	Reference			
	> 60	73	2.0 (1.2–3.3)	**0.009**	2.3 (1.4–4.0.4.0)	**0.002**
**Gende**r	Female	45	Reference			
	Male	120	0.9 (0.5–1.5)	0.784		
**Plasma CEA**	≤ 10 µg/L	133	Reference			
	> 10 µg/L	32	0.7 (0.4–1.4)	0.303		
**Primary cancer**						
**Location**	Left	146	Reference			
	Right	19	2.5 (1.3–5.0.3.0)	**0.008**	2.5 (1.2–5.2)	**0.011**
**Pathologic tumor category**	ypT0 – ypT3	136	Reference			
	ypT4	29	1.7 (0.9–3.1)	0.125	1.7 (0.9–3.3)	0.110
**Pathologic lymphatic spread**	Negative	57				
	Positive	108	1.2 (0.7–2.0.7.0)	0.588		
**Extrahepatic metastasis**	No	122	Reference			
	Yes	43	1.5 (0.9–2.7)	0.145	1.8 (1.0–3.2.0.2)	**0.055**
**Initial CRLM**						
Number	≤ 2	86	Reference			
	> 2	79	1.7 (1.0–2.8.0.8)	0.049	1.2 (0.5–3.0.5.0)	0.700
Maximum diameter	≤ 3 cm	124	Reference			
	> 3 cm	43	0.9 (0.5–1.8)	0.816		
**Ablated CRLM**						
Number	≤ 2	114	Reference			
	> 2	51	1.5 (0.9–2.6)	0.118	1.3 (0.6–2.7)	0.550
Maximum diameter	≤ 16 mm	112	Reference			
	> 16 mm, ≤ 30 mm	53	0.8 (0.4–1.5)	0.492		
Distribution	unilobar	113	Reference			
	bilobar	52	1.2 (0.7–1.9)	0.597		
**DLM**	No	97	Reference			
	Yes	68	1.5 (0.9–2.5)	0.114	1.2 (0.6–2.5)	0.590
**Timing of MWA for sCRLM**	Bowel-first	64	Reference			
	Liver-first	32	0.7 (0.4–1.4)	0.344		
	Concurrent	69	0.7 (0.4–1.2)	0.226		

*DLM* Disappearing colorectal liver metastases. *CRLM* Colorectal liver metastases. *CEA* Carcinoembryonic antigen

## Discussion

Pre-ablative chemotherapy commonly results in DLM, making it challenging to eradicate micro metastases at DLM sites. Gaining insight into how DLM affects the outcomes of percutaneous MWA is crucial for determining the optimal timing for MWA in conjunction with systemic therapy. Our findings demonstrate that patients with unablated DLM are associated with poorer ihPFS than those without DLM, although it does not significantly influence OS.

It is noteworthy that radiographic identified DLM does not necessarily correspond to a pathologically complete remission, with approximately 42.3% to 50% of the DLM detected on preoperative CT showed pathologically viable cancer cells or recurred during the follow-up [9, 10]. Studies using MRI reported complete remission at DLM sites occurred in 39%−85% of cases [11, 12]. These undetected tumors might still be viable but could not be detected and ablated for percutaneous MWA. Therefore, it resulted in a high recurrence rate at one-third of DLM sites and worse ihPFS rates for patients with DLM in the present study.

However, the difference of OS was not statistically significant between patients with or without DLM in our study. These results were consistent with a systematic review showing that patients with resected DLM experienced longer disease-free survival compared to those with DLM left in situ, although differences in OS were not observed [9]. We suppose that the reasons for comparable OS for patients with DLM may lie in their favorable tumor biology with better response to chemotherapy. Moreover, most tumor recurrences can be re-treated with radical local treatment, potentially leading to comparable OS outcomes of patients with DLM. In addition, the median follow-up of 48.2 months may be insufficient to demonstrate OS distinction.

DLM refers to lesion visible on baseline CEMRI that disappears following chemotherapy [18]. After chemotherapy, the sensitivity of CEMRI, CEUS, CECT and PET/CT for diagnosing CRLM was 90%, 79%, 77% and 48%, respectively, whereas CEMRI showed significantly highest sensitivity and was recommended as first choice for patients receiving chemotherapy [19]. Most institutions use CECT only for assessment due to its ability to complete evaluation for intrahepatic metastases and extrahepatic disease. However, the sensitivity of CECT in patients with chemotherapy was significantly worse than those without (77% vs. 91%, *P* = 0.024) [19], leading to elevated observation rates of DLM [20, 21]. Intraoperative CEUS (IOCEUS) proved equivalent in sensitivity to MRI (95% and 90%). An additional 40% and 46% of tumors invisible on preoperative imaging might be detected using IOCEUS [22, 23]. Regrettably, IOCEUS can only be performed during the operation. Radiographic DLM is currently managed conservatively by regular follow-up for patients underwent percutaneous MWA in our center. One primary limitation of imaging-guided percutaneous MWA is that current imagings are powerless to detect all residual lesions. To compensate this limitation, we used high-frequency US and CEUS to maximize the CRLM detection and guide ablation. Our previous study demonstrated that application of trans-abdominal high-frequency US and CEUS could significantly improve the detection rate of superficial lesions and small CRLM than convex probe CEUS, especially for patients after chemotherapy [15].

The optimal strategy for employing thermal ablation combined with systemic therapy in CRLM is under debate. In our previous study, we investigated how the timing of ablation during chemotherapy affects oncologic outcomes and revealed that disease-free survival or progression-free survival were significantly worse in delayed versus the upfront MWA groups [24, 25], suggesting to early and completely ablate tumors. We believe that chemotherapy-induced liver damage and tumor shrinkage may obscure tumor margins, resulting in inaccurate antenna positions under imaging guidance, contributing to incomplete ablation. In the present study, patients with DLM received more cycles of pre-ablative chemotherapy, resulting in a delay in the timing of MWA compared to those without DLM. Our finding also demonstrated DLM as a risk factor for ihPFS after MWA. Given that DLM is frequently associated with systemic therapy and tends to lead to a higher rate of intrahepatic recurrence, it is reasonable to speculate that radiological DLM during delayed ablation contributes to the decrease in disease-free survival as compared to up-front ablation, which eliminates tumor early and completely. Therefore, we advocate for a more proactive approach to eliminate tumor early and completely, ideally before they become radiographic undetectable. This strategy may help reduce the risk of recurrence, enhance patient outcomes, minimize the need for repeated local interventions, and subsequently conserve medical resources.

This study has several limitations. Firstly, the retrospective design led to variability in the imaging modalities used to identify DLM, as not all patients underwent MRI examinations before and after chemotherapy. However, despite this variability, the patients with DLM in this study represent those who could not achieve NED through ultrasound-guided percutaneous MWA. Thus, whether CT or MRI was used to define DLM is unlikely to affect the results. Secondly, the chemotherapy regimens and durations varied among patients. As chemotherapy protocols have evolved over the study period, some patients received targeted biological therapies in addition to chemotherapy. Lastly, patients with DLM or disease progression who opted for supportive care instead of local treatment were excluded from the analysis, which may have introduced selection bias. Lastly, patients with new lesions during chemotherapy were excluded from the analysis, which may have introduced selection bias. It is reported that patients with one or more new lesions at disease progression were associated with significantly worse survival [26]. Including these patients in the group without DLM could confound the results.

In conclusion, patients with DLM presented worse ihPFS, suggesting that to eradicate visible tumors before disappearance may be advantageous when synchronous CRLM is ablatable.

## Data Availability

No datasets were generated or analysed during the current study.

## References

[1] Siriwardena AK, Mason JM, Mullamitha S, et al. Management of colorectal cancer presenting with synchronous liver metastases. Nat Rev Clin Oncol. 2014;11:446–59. 24889770 10.1038/nrclinonc.2014.90

[2] Ghiasloo M, Pavlenko D, Verhaeghe M, et al. Surgical treatment of stage IV colorectal cancer with synchronous liver metastases: a systematic review and network meta-analysis. Eur J Surg Oncol. 2020;46:1203–13. 32178961 10.1016/j.ejso.2020.02.040

[3] Martin J, Petrillo A, Smyth EC, et al. Colorectal liver metastases: current management and future perspectives. World J Clin Oncol. 2020;11:761–808. 33200074 10.5306/wjco.v11.i10.761 PMC7643190

[4] Nordlinger B, Sorbye H, Glimelius B, et al. Perioperative chemotherapy with FOLFOX4 and surgery versus surgery alone for resectable liver metastases from colorectal cancer (EORTC intergroup trial 40983): a randomised controlled trial. Lancet. 2008;371:1007–16. 18358928 10.1016/S0140-6736(08)60455-9 PMC2277487

[5] Nordlinger B, Sorbye H, Glimelius B, et al. Perioperative FOLFOX4 chemotherapy and surgery versus surgery alone for resectable liver metastases from colorectal cancer (EORTC 40983): long-term results of a randomised, controlled, phase 3 trial. Lancet Oncol. 2013;14:1208–15. 24120480 10.1016/S1470-2045(13)70447-9

[6] Bischof DA, Clary BM, Maithel SK, et al. Surgical management of disappearing colorectal liver metastases. Br J Surg. 2013;100:1414–20. 24037559 10.1002/bjs.9213

[7] Kuhlmann K, van Hilst J, Fisher S, et al. Management of disappearing colorectal liver metastases. Eur J Surg Oncol. 2016;42:1798–805. 27260846 10.1016/j.ejso.2016.05.005

[8] Adam R, Delvart V, Pascal G et al. Rescue surgery for unresectable colorectal liver metastases downstaged by chemotherapy: a model to predict long-term survival. Ann Surg 2004;240:644 – 57; discussion 57 – 8. 10.1097/01.sla.0000141198.92114.f6 PMC1356466 15383792

[9] Barimani D, Kauppila JH, Sturesson C, et al. Imaging in disappearing colorectal liver metastases and their accuracy: a systematic review. World J Surg Oncol. 2020;18:264. 33032620 10.1186/s12957-020-02037-w PMC7545848

[10] Nassar A, Cimpean S, Abdelhamid A, et al. The dilemma of the disappeared colorectal liver metastasis: systematic review of reviews and evidence gap map. BJS Open. 2022. 10.1093/bjsopen/zrac051. 35598157 10.1093/bjsopen/zrac051 PMC9124362

[11] Owen JW, Fowler KJ, Doyle MB, et al. Colorectal liver metastases: disappearing lesions in the era of Eovist hepatobiliary magnetic resonance imaging. HPB (Oxford). 2016;18:296–303. 27017170 10.1016/j.hpb.2015.10.009 PMC4814600

[12] Kim SS, Song KD, Kim YK, et al. Disappearing or residual tiny (≤ 5 mm) colorectal liver metastases after chemotherapy on Gadoxetic acid-enhanced liver MRI and diffusion-weighted imaging: is local treatment required? Eur Radiol. 2017;27:3088–96. 27815722 10.1007/s00330-016-4644-4

[13] Ruers T, Punt C, Van Coevorden F, et al. Radiofrequency ablation combined with systemic treatment versus systemic treatment alone in patients with non-resectable colorectal liver metastases: a randomized EORTC intergroup phase II study (EORTC 40004). Ann Oncol. 2012;23:2619–26. 22431703 10.1093/annonc/mds053 PMC3457746

[14] Ruers T, Van Coevorden F, Punt CJ, et al. Local treatment of unresectable colorectal liver metastases: results of a randomized phase II trial. J Natl Cancer Inst. 2017;109(9):djx015. 10.1093/jnci/djx015. 10.1093/jnci/djx015 PMC5408999 28376151

[15] Qin S, Chen Y, Liu XY, et al. Clinical application of contrast-enhanced ultrasound using high-frequency linear probe in the detection of small colorectal liver metastases. Ultrasound Med Biol. 2017;43:2765–73. 29037844 10.1016/j.ultrasmedbio.2017.08.932

[16] Qin S, Liu GJ, Huang M, et al. The local efficacy and influencing factors of ultrasound-guided percutaneous microwave ablation in colorectal liver metastases: a review of a 4-year experience at a single center. Int J Hyperthermia. 2019;36:36–43. 30489175 10.1080/02656736.2018.1528511

[17] Qin S, Hu H, Cui R, et al. A prognostic nomogram for intrahepatic progression-free survival in patients with colorectal liver metastases after ultrasound-guided percutaneous microwave ablation. Int J Hyperthermia. 2022;39:144–54. 35012413 10.1080/02656736.2021.2023226

[18] Siriwardena AK, Serrablo A, Fretland AA, et al. Multisocietal European consensus on the terminology, diagnosis, and management of patients with synchronous colorectal cancer and liver metastases: an E-AHPBA consensus in partnership with ESSO, ESCP, ESGAR, and CIRSE. Br J Surg. 2023;110:1161–70. 37442562 10.1093/bjs/znad124 PMC10416695

[19] Rojas Llimpe FL, Di Fabio F, Ercolani G, et al. Imaging in resectable colorectal liver metastasis patients with or without preoperative chemotherapy: results of the PROMETEO-01 study. Br J Cancer. 2014;111:667–73. 24983362 10.1038/bjc.2014.351 PMC4134499

[20] Tanaka K, Takakura H, Takeda K, et al. Importance of complete pathologic response to prehepatectomy chemotherapy in treating colorectal cancer metastases. Ann Surg. 2009;250:935–42. 19953712 10.1097/sla.0b013e3181b0c6e4

[21] van Vledder MG, de Jong MC, Pawlik TM, et al. Disappearing colorectal liver metastases after chemotherapy: should we be concerned? J Gastrointest Surg. 2010;14:1691–700. 20839072 10.1007/s11605-010-1348-y

[22] Arita J, Ono Y, Takahashi M, et al. Usefulness of contrast-enhanced intraoperative ultrasound in identifying disappearing liver metastases from colorectal carcinoma after chemotherapy. Ann Surg Oncol. 2014;21(Suppl 3):S390–7. 24570378 10.1245/s10434-014-3576-y

[23] Tani K, Shindoh J, Akamatsu N, et al. Management of disappearing lesions after chemotherapy for colorectal liver metastases: relation between detectability and residual tumors. J Surg Oncol. 2018;117:191–7. 28876456 10.1002/jso.24805

[24] Li J, Liu G, Xie X, et al. Outcomes following different thermal ablation strategies in patients with unresectable colorectal liver metastases. Radiology. 2023;308:e223135. 37581502 10.1148/radiol.223135

[25] Li J, Yu J, Liu G, et al. Up-front versus delayed thermal ablation for colorectal liver oligometastases: a multicenter retrospective study using propensity score matching. AJR Am J Roentgenol. 2023;220:885–99. 36516005 10.2214/AJR.22.28603

[26] Hall PE, Shepherd STC, Brown J, et al. Radiological response heterogeneity is of prognostic significance in metastatic renal cell carcinoma treated with vascular endothelial growth factor-targeted therapy. Eur Urol Focus. 2020;6:999–1005. 30738795 10.1016/j.euf.2019.01.010

